# Supplementary material for: Development and psychometric properties of maternal health literacy inventory in pregnancy
Source: PLoS One. 2020 Jun 11;15(6):e0234305. doi: 10.1371/journal.pone.0234305 (PMC7289409; doi:10.1371/journal.pone.0234305)
Supplement: S1 File — (DOCX) [file pone.0234305.s001.docx]

Consent to participate in the project ‘Design and Psychometric Assessment of a Prenatal Maternal Health Literacy Assessment Tool: A Sequential Combined Exploratory Study’

**Dear Mrs**

You are invited to participate in the above-mentioned research. Information about this research is provided in this service sheet and you are free to participate or not to participate in this research.

You do not have to make an immediate decision, and you can ask your research team questions and consult with anyone you want. Before signing this consent form, make sure you understand all the information on this form and that all your questions have been answered.

**Research Executive**

**1.I understand that the aims of this study are:**

Designing a Maternal Health Literacy Assessment Questionnaire in Pregnancy and Verifying it for Measuring Maternal Health Literacy in Pregnancy

**2.I know that my participation in this research is completely voluntary and I do not have to**

I was assured that if I did not participate in this study, I would not be deprived of routine diagnostic and therapeutic care, and that my treatment relationship with the treatment center and physician would not be impaired.

**3**.**I know that even after agreeing to participate in the research, I can withdraw from the research at any time, after informing the reviewer**, and my withdrawal from the research will not deprive me of the usual medical care.

**4.The way I collaborate in this research is as follows:** First, the goals of this project are explained and then I complete the designed questionnaire. I have been told that it may take 10 to 20 minutes to complete the questionnaire.

**5.The potential benefits of participating in this study are as follows:**

At the end of the questionnaire, the researcher will answer if I have any questions regarding pregnancy and postpartum. By participating in this project, I hope to get information that will help me improve my pregnancy status and outcomes.

**6.Potential damages and complications of this study are as follows:**

No harm or complication threatens me.

8.**If I do not want to participate in the study, I will be offered the usual treatment, with the benefits and side effects of this:** The usual services provided at the health center in full and without any complications.

**9**.**I understand that the authors of this research have kept all information about me confidential** and are only permitted to publish only general and collective results of this research without mentioning my name or profile.

**10.I know that the Research Ethics Committee can access my information** in order to monitor my rights.

11.**I know that I will not bear the costs of any research interventions.**

Dr. Ziba Taghizadeh was introduced to answer my questions and I was told to contact him whenever any questions regarding participation in the study.

**Address**: Faculty of Nursing and Midwifery, Tohid Square, Tehran, Iran

**Phone: 989122147081**

**989122147081: Mobile**

1.I understand that if any and every problem, both physical and mental, arises during the course of my research, due to my participation in this research, I will be treated for complications, costs, and associated compensation.

2.I know that if I have any objections to the researcher or the research process, I can contact the Research Ethics Committee of Tehran University of Medical Sciences at: Tehran University of Medical Sciences Central Building, Keshavarz Avenue., Call Room 501 and report your problem orally or in writing.

3.This form of information and informed consent will be arranged in two copies and after signing one copy will be provided to me and the other to the presenter.

I read and understood the consents items, I hereby declare my informed consent to participate in this research.

**Signature of the participant**

I commit myself to enforce my obligations under the above provisions and undertake to endeavor to ensure the rights and safety of the participants in this study.

**Research Executive's signature**
