# Supplementary material for: Development and psychometric properties of maternal health literacy inventory in pregnancy
Source: PLoS One. 2020 Jun 11;15(6):e0234305. doi: 10.1371/journal.pone.0234305 (PMC7289409; doi:10.1371/journal.pone.0234305)
Supplement: S2 File — (DOCX) [file pone.0234305.s002.docx]

**Questions guide in Qualitative phase**

In Qualitative phase, the interview guide included the following questions that were identified as the progress of the study and data collection, the simultaneous analysis and the creation of subcategories of the next interview route.

1. What did you want to know when you became pregnant? What information do you need? What information do you think a pregnant woman should have? Need to have?

2. Who did you get this information from, where did you find it to be true, relevant, relevant to what you were doing, please explain to me? Please tell me about your experience?

3. probing Questions: Can you explain more about this? Can you give an example?
