# Supplementary material for: Development and psychometric properties of maternal health literacy inventory in pregnancy
Source: PLoS One. 2020 Jun 11;15(6):e0234305. doi: 10.1371/journal.pone.0234305 (PMC7289409; doi:10.1371/journal.pone.0234305)
Supplement: S4 File — (DOCX) [file pone.0234305.s004.docx]

**The maternal health literacy inventory in pregnancy (MHELIP(**

| **Item** | **I don't know at all** | **I know a little** | **I know something** | **I know a lot** | **I know fully** |
| --- | --- | --- | --- | --- | --- |
| 1.I know natural physical changes during pregnancy. |  |  |  |  |  |
| 2.I know natural psychological changes during pregnancy. |  |  |  |  |  |
| 3.I know proper nutrition during pregnancy. |  |  |  |  |  |
| 4.I know personal health care. |  |  |  |  |  |
| 5.I know Proper activity and status in pregnancy. |  |  |  |  |  |
| 6.I know proper exercise during pregnancy. |  |  |  |  |  |
| 7.I know pregnancy supplements (vitamins). |  |  |  |  |  |
| 8.I know the appropriate referral timing for pregnancy examinations (visits). |  |  |  |  |  |
| 9.I know diagnostic examination (ultrasound and tests) of maternal and fetal health in pregnancy. |  |  |  |  |  |
| 10.I know the acceptable and normal amount of weight gain during pregnancy. |  |  |  |  |  |
| 11.I know common pregnancy problems such as nausea, vomiting, lower back pain. |  |  |  |  |  |
| 12.I know injecting safe (allowed) vaccines during pregnancy. |  |  |  |  |  |
| 13.I know the proper sexual relation during pregnancy. |  |  |  |  |  |
| 14.I know the normal number of fetal movements. |  |  |  |  |  |
| 15.I know the factors affecting fetal health such as photography, medications, chemicals such as botox, etc . |  |  |  |  |  |
| 16.I know risk signs in pregnancy. |  |  |  |  |  |
| 17.I know pregnancy disease symptoms such as gestational diabetes, high blood pressure in pregnancy and other diseases. |  |  |  |  |  |
| 18.I know childbirth such as the advantages and disadvantages of each of the natural delivery methods and cesarean section and their associated care. |  |  |  |  |  |
| 19.I know the methods of pain relief in virginal delivery. |  |  |  |  |  |
| 20.I know neonatal and infant care in the postpartum period. |  |  |  |  |  |
| 21.I know required postpartum care of mother. |  |  |  |  |  |
| **Item** | **Not at all** | **rarely** | **sometimes** | **most often the times** | **always** |
| 22.I acquire information from written materials such as books, educational notes, pamphlets and medication brochures. |  |  |  |  |  |
| 23.I acquire information from radio and television. |  |  |  |  |  |
| 24.I acquire information from internet sources such as websites, instagram and telegram . |  |  |  |  |  |
| 25.I acquire information from other pregnant women. |  |  |  |  |  |
| 26.I acquire information from family, friends and acquaintances. |  |  |  |  |  |
| 27.I acquire information from healthcare professionals such as a physician or midwife. |  |  |  |  |  |
| 28.It is easy for me to read and pronounce pregnancy-related vocabulary from information sources such as books, educational booklets, internet, telegram and Instagram. |  |  |  |  |  |
| 29.The information obtained from different sources of information are understandable for me. |  |  |  |  |  |
| 30.I know valid and verified sources for getting the right pregnancy related information. |  |  |  |  |  |
| 31. I ask of the doctor or midwife to make sure pregnancy related information. |  |  |  |  |  |
| 32.I Evaluate the accuracy of pregnancy-related information obtained from online sources such as websites, instagram and telegram. |  |  |  |  |  |
| 33.I Evaluate the accuracy of pregnancy-related information obtained from friends and relatives |  |  |  |  |  |
| 34. I able to control/management physical and psychological changes in pregnancy. |  |  |  |  |  |
| 35.I implement a proper diet for pregnancy. |  |  |  |  |  |
| 36.I implement necessary measures for personal health care during pregnancy. |  |  |  |  |  |
| 37.I adhere to the principles of activity and proper condition during pregnancy. |  |  |  |  |  |
| 38.I take pregnancy supplements as prescribe by doctor or midwife. |  |  |  |  |  |
| 39.I consult with the doctor or midwife for taking any type of medication during pregnancy (chemical and herbal). |  |  |  |  |  |
| 40.I attend for prenatal care (examinations) as scheduled. |  |  |  |  |  |
| 41.I Perform ultrasound and tests in pregnancy recommended by healthcare professionals such as doctor or midwife. |  |  |  |  |  |
| 42.I monitor and control the weight gain during pregnancy. |  |  |  |  |  |
| 43.I use the appropriate methods of sexual relation during pregnancy. |  |  |  |  |  |
| 44.I avoid taking actions that are harmful to pregnancy. |  |  |  |  |  |
| 45.I see the doctor or midwife as soon as possible when any signs of danger in pregnancy is observed. |  |  |  |  |  |
| 46.I ask the doctor or midwife for further explanation if the information and recommendations are not clear enough. |  |  |  |  |  |
| 47.I participate in decision making about pregnancy issues with the doctor or midwife (providing personal opinions). |  |  |  |  |  |
| 48.I pay attention to the accuracy and appropriateness of information given to other pregnant women. |  |  |  |  |  |
| **Maternal Health Knowledge**: 1-21 question  **Search for maternal health information**: 22-27 question  **Assessment of Maternal Health Information**:28-33 question  **Maternal Health Decision Making and Behavior**:34-48 question | | | | | |

**©** Taheri S. et al., 2018
