# Supplementary material for: Development and psychometric properties of maternal health literacy inventory in pregnancy
Source: PLoS One. 2020 Jun 11;15(6):e0234305. doi: 10.1371/journal.pone.0234305 (PMC7289409; doi:10.1371/journal.pone.0234305)
Supplement: S5 File — (DOC) [file pone.0234305.s005.doc]

**Manual for scoring the maternal health literacy inventory in pregnancy (MHELIP (**

|  | **Number of items** | **Minimum possible raw score** | **Maximum possible raw score** |
| --- | --- | --- | --- |
| **Maternal Health Knowledge** | **21 (item 1-21)** | **21** | **105** |
| **Search for maternal health information** | **6 (item 22-27)** | **6** | **30** |
| **Assessment of Maternal Health Information** | **6 (item 28-33)** | **6** | **30** |
| **Maternal Health Decision Making and Behavior** | **15(item 34-48)** | **15** | **75** |

**To calculate each subscale or total score for the MHELIP, first we added raw scores and linearly transferred it to a score from 0 to 100 using the following formula.**

**Score=
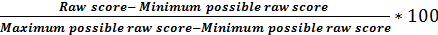
**

**We ranked the MHELIP score to 4** **categories: ‘inadequate’,** **‘problematic’ (which together also define ‘limited’ health literacy),** **‘sufficient and** **‘excellent’ (which together also defined ‘desired’ health literacy):**

**Inadequate= 0–50**

**Problematic= 50.1–66**

**Sufficient= 66.1–84**

**Excellent=84.1-100**

**©** Taheri S. et al., 2018
