# Supplementary material for: Development and psychometric properties of maternal health literacy inventory in pregnancy
Source: PLoS One. 2020 Jun 11;15(6):e0234305. doi: 10.1371/journal.pone.0234305 (PMC7289409; doi:10.1371/journal.pone.0234305)
Supplement: Attachment — Submitted filename: PONE-D-19-24536_review.pdf [file pone.0234305.s006.pdf]

PLOS ONE

Development and Psychometric Properties of maternal health literacy inventory in pregnancy (MHELIP)

--Manuscript Draft--

**Manuscript Number:**	PONE-D-19-24536
**Article Type:**	Research Article
**Full Title:**	Development and Psychometric Properties of maternal health literacy inventory in pregnancy (MHELIP)
**Short Title:**	maternal health literacy inventory in pregnancy
**Corresponding Author:**	ziba taghizadeh, Phd<br>Tehran University of Medical Sciences<br>Tehran, IRAN, ISLAMIC REPUBLIC OF
**Keywords:**	Maternal health literacy - Pregnancy - Psychometric - Questionnaire design
**Abstract:**	Background<br><br>Pregnancy is one of the most sensitive and important stages of women's life. Maternal health literacy is the key to achieving a healthy pregnancy. It also affects pregnancy outcomes by improving the quality of health care in this period. The purpose of this study was to development and psychometric properties a tool for measuring maternal health literacy during pregnancy.Methods<br><br>This sequential, exploratory and mixed study was carried out in two parts (qualitative study and psychometric evaluation of the tool) in Tehran in 2016-18. The first part involved a qualitative content analysis with a traditional approach using in-depth personal interviews with 19 eligible pregnant women. Then, the pool of items extracted from the qualitative part was completed by reviewing the existing literature and tools. In the second part, the overlapping items were summarized and the tool was validated. In order to evaluate the construct validity, a cross-sectional study was conducted with the participation of 320 pregnant women. Data analysis was performed by SPSS-19 software using exploratory factor analysis and reliability tests (Cronbach's alpha and ICC).Results<br><br>Findings of qualitative part produced a pool of 120 items that reached to 124 items after reviewing the literature. After confirming face and content validity by calculating CVI and CVR for each item, 53 items remained. Finally, the results of exploratory factor analysis confirmed a tool with 48 items in four factors, explaining 46.49% of the variance of total variables of the tool. In order to confirm the stability of the tool, the internal consistency of 4 factors was 0.94 using Cronbach's alpha and their intra-cluster correlation was 0.96 using test-retest method two weeks later. Finally, the tool was finalized with 48 items in 4 dimensions, including "Maternal Health Knowledge", "Maternal Health Information Search", "Maternal Health Information Assessment" and "Maternal Health Decision Making and Behavior".Conclusion<br><br>The designed tool is a multidimensional, reliable and validated scale for assessing maternal health literacy during pregnancy. It also can address different dimensions of maternal health literacy in pregnant women by considering their experiences in a qualitative study.
**Order of Authors:**	ziba taghizadeh, Phd
	safoura taheri
	Mahmoud Tavousi
	zohre momenimovahed
	nasibe sharifi
	azita tiznobaik

Powered by Editorial Manager® and ProduXion Manager® from Aries Systems Corporation

	Zainab Suhrabi
**Additional Information:**	
**Question**	**Response**
**Financial Disclosure**<br><br>Enter a financial disclosure statement that describes the sources of funding for the work included in this submission. Review the submission guidelines for detailed requirements. View published research articles from *PLOS ONE* for specific examples.<br><br>This statement is required for submission and **will appear in the published article** if the submission is accepted. Please make sure it is accurate.<br><br>**Unfunded studies**<br>Enter: *The author(s) received no specific funding for this work.*<br><br>**Funded studies**<br>Enter a statement with the following details:<br>• Initials of the authors who received each award<br>• Grant numbers awarded to each author<br>• The full name of each funder<br>• URL of each funder website<br>• Did the sponsors or funders play any role in the study design, data collection and analysis, decision to publish, or preparation of the manuscript?<br>• **NO** - Include this sentence at the end of your statement: *The funders had no role in study design, data collection and analysis, decision to publish, or preparation of the manuscript.*<br>• **YES** - Specify the role(s) played.<br><br>* typeset	The funders had no role in study design, data collection and analysis, decision to publish, or preparation of the manuscript.
**Competing Interests**<br><br>Use the instructions below to enter a competing interest statement for this submission. On behalf of all authors, disclose any competing interests that could be perceived to bias this work—acknowledging all financial support and any other relevant financial or non-financial competing interests.	The authors have declared that no competing interests exist.

This statement **will appear in the published article** if the submission is accepted. Please make sure it is accurate. View published research articles from *PLOS ONE* for specific examples.<br><br>**NO authors have competing interests**<br><br>Enter: *The authors have declared that no competing interests exist.*<br><br>**Authors with competing interests**<br><br>Enter competing interest details beginning with this statement:<br><br>*I have read the journal's policy and the authors of this manuscript have the following competing interests: [insert competing interests here]*<br><br>* typeset	
**Ethics Statement**<br><br>Enter an ethics statement for this submission. This statement is required if the study involved:<br><br>• Human participants<br>• Human specimens or tissue<br>• Vertebrate animals or cephalopods<br>• Vertebrate embryos or tissues<br>• Field research<br><br>Write "N/A" if the submission does not require an ethics statement.<br><br>General guidance is provided below. Consult the submission guidelines for detailed instructions. **Make sure that all information entered here is included in the Methods section of the manuscript.**	In order to comply with ethical considerations, permission was obtained from the Faculty of Nursing and Midwifery of Tehran University of Medical Sciences and the Deputy for Research (IR.TUMS.VCR.REC.1395.1866). Written consent was obtained for the participants and confidentiality of their personal information was maintained at all stages of the study.

Powered by Editorial Manager® and ProduXion Manager® from Aries Systems Corporation

**Format for specific study types**<br><br>**Human Subject Research (involving human participants and/or tissue)**<br>• Give the name of the institutional review board or ethics committee that approved the study<br>• Include the approval number and/or a statement indicating approval of this research<br>• Indicate the form of consent obtained (written/oral) or the reason that consent was not obtained (e.g. the data were analyzed anonymously)<br><br>**Animal Research (involving vertebrate animals, embryos or tissues)**<br>• Provide the name of the Institutional Animal Care and Use Committee (IACUC) or other relevant ethics board that reviewed the study protocol, and indicate whether they approved this research or granted a formal waiver of ethical approval<br>• Include an approval number if one was obtained<br>• If the study involved *non-human primates*, add *additional details* about animal welfare and steps taken to ameliorate suffering<br>• If anesthesia, euthanasia, or any kind of animal sacrifice is part of the study, include briefly which substances and/or methods were applied<br><br>**Field Research**<br><br>Include the following details if this study involves the collection of plant, animal, or other materials from a natural setting:<br>• Field permit number<br>• Name of the institution or relevant body that granted permission	
**Data Availability**<br><br>Authors are required to make all data underlying the findings described fully available, without restriction, and from the time of publication. PLOS allows rare exceptions to address legal and ethical concerns. See the PLOS Data Policy and FAQ for detailed information.	No - some restrictions will apply

Powered by Editorial Manager® and ProduXion Manager® from Aries Systems Corporation

A Data Availability Statement describing where the data can be found is required at submission. Your answers to this question constitute the Data Availability Statement and **will be published in the article**, if accepted.<br><br>**Important:** Stating 'data available on request from the author' is not sufficient. If your data are only available upon request, select 'No' for the first question and explain your exceptional situation in the text box.<br><br>Do the authors confirm that all data underlying the findings described in their manuscript are fully available without restriction?	
**Describe where the data may be found in full sentences. If you are copying our sample text, replace any instances of XXX with the appropriate details.**<br><br>• If the data are **held or will be held in a public repository**, include URLs, accession numbers or DOIs. If this information will only be available after acceptance, indicate this by ticking the box below. For example: *All XXX files are available from the XXX database (accession number(s) XXX, XXX.)*.<br>• If the data are all contained **within the manuscript and/or Supporting Information files**, enter the following: *All relevant data are within the manuscript and its Supporting Information files.*<br>• If neither of these applies but you are able to provide **details of access elsewhere**, with or without limitations, please do so. For example:<br><br>*Data cannot be shared publicly because of [XXX]. Data are available from the XXX Institutional Data Access / Ethics Committee (contact via XXX) for researchers who meet the criteria for access to confidential data.*<br><br>*The data underlying the results presented in the study are available from (include the name of the third party*	The datasets used and/or analyzed during the current study are available from the corresponding author on reasonable request.

and contact information or URL).<br>• This text is appropriate if the data are owned by a third party and authors do not have permission to share the data.<br><br>* typeset	
Additional data availability information:	

Powered by Editorial Manager® and ProduXion Manager® from Aries Systems Corporation

Click here to download Manuscript Taheri -MHELIP.doc

Development and Psychometric Properties of maternal health literacy inventory in pregnancy (MHELIP)

Safoura Taheri[1], Mahmoud Tavousi[2], zohre momenimovahed[3], nasibe sharifi[1], azita tiznobaik[4], Zainab Suhrabi [1], Ziba Taghizadeh[5*]

1. Department of midwifery, School of Nursing & Midwifery, Ilam University of Medical Sciences, Ilam, Iran

2. Health Metrics Research Center, Iranian Institute for Health Sciences Research, ACECR, Tehran, Iran.

3. Department of Midwifery, School of Nursing and Midwifery, Qom University of Medical Sciences, Qom, Iran.

4.Department of Midwifery, Faculty of Nursing and Midwifery, Member of Mother and Child Care Research Center, Hamadan University of Medical Sciences and Health Services, Hamadan, Iran.

5. Department of Midwifery and Reproductive Health, School of Nursing and Midwifery, Tehran University of Medical Sciences, Tehran, Iran.

Authors' E-mails:

SA: taheri_safura@yahoo.com

MT: tavousi@acecr.ac.ir

ZM: momeni.z@gmail.com

N SH: nasibe.sharifi@yahoo.com

AT: azita.tizno@gmail.com

MJ: Suhrabi2007@yahoo.com

ZT: zibataghizadeh@yahoo.com

Address for correspondence:

Dr Ziba Taghizadeh, Department of Midwifery and Reproductive Health, School of Nursing and Midwifery, Tehran University of Medical Sciences, East Nosrat St., Tehran, Iran.

Tel:++98-21-61054203

E-mail: zibataghizadeh@yahoo.com

Abstract

Background: Pregnancy is one of the most sensitive and important stages of women's life. Maternal health literacy is the key to achieving a healthy pregnancy. It also affects pregnancy outcomes by improving the quality of health care in this period. The purpose of this study was to development and psychometric properties a tool for measuring maternal health literacy during pregnancy.

Methods: This sequential, exploratory and mixed study was carried out in two parts (qualitative study and psychometric evaluation of the tool) in Tehran in 2016-18. The first part involved a qualitative content analysis with a traditional approach using in-depth personal interviews with 19 eligible pregnant women. Then, the pool of items extracted from the qualitative part was completed by reviewing the existing literature and tools. In the second part, the overlapping items were summarized and the tool was validated. In order to evaluate the construct validity, a cross-sectional study was conducted with the participation of 320 pregnant women. Data analysis was performed by SPSS-19 software using exploratory factor analysis and reliability tests (Cronbach's alpha and ICC).

Results: Findings of qualitative part produced a pool of 120 items that reached to 124 items after reviewing the literature. After confirming face and content validity by calculating CVI and CVR for each item, 53 items remained. Finally, the results of exploratory factor analysis confirmed a tool with 48 items in four factors, explaining 46.49% of the variance of total variables of the tool. In order to confirm the stability of the tool, the internal consistency of 4 factors was 0.94 using Cronbach's alpha and their intra-cluster correlation was 0.96 using test-retest method two weeks later. Finally, the tool was finalized with 48 items in 4 dimensions, including "Maternal Health Knowledge", "Maternal Health Information Search", "Maternal Health Information Assessment" and "Maternal Health Decision Making and Behavior".

Conclusion: The designed tool is a multidimensional, reliable and validated scale for assessing maternal health literacy during pregnancy. It also can address different dimensions of maternal health literacy in pregnant women by considering their experiences in a qualitative study.

Keywords: Maternal health literacy - Pregnancy - Psychometric - Questionnaire design

Introduction

Pregnancy is one of the most sensitive and important stages of women's life. The importance of pregnancy is significant because the health and well-being of mother directly affect the life of another person (1, 2). According to the World Health Organization (WHO) 2010 report, approximately 1,000 pregnant women worldwide die every day due to pregnancy and childbirth complications, with 99% of them occurring in developing countries (3).

Early onset of prenatal care in the first trimester of pregnancy and its continuation during pregnancy will lead to improved pregnancy and delivery outcomes. Careful prenatal care will prevent maternal-fetal complications (4). However, despite the provision of different forms of care by health care providers, some factors appear to prevent the proper and timely delivery of care during pregnancy. One of these factors is the lack of maternal health literacy during pregnancy (5).

Maternal health literacy refers to cognitive and social skills that motivate and enable women to acquire, understand, and use information in a way that safeguards and promotes their health and health of their children (6). Health literacy is the key to achieving a healthy motherhood and has an impact on pregnancy outcomes by improving the quality of health care during pregnancy (7). Maternal health literacy empowers women to receive timely prenatal, decision making, and labor management education such as accepting midwifery interventions and even pain management (8, 9). In a qualitative study that examined the understanding and perception of pregnant women about health literacy (pregnancy risk symptoms, preparation for childbirth and its complications, and understanding of neonatal care), the results showed that women were aware of the risks of pregnancy and infancy, but they were weak in interpreting and applying the key information provided during prenatal care (10). In a study in Tanzania, 42% of pregnant women (11) and in a study in Haun (2014) about 30% of mothers did not know any of the signs of risk in pregnancy and childbirth (12).

It should be noted that because health literacy has a variety of definitions and structures, researchers have designed different tools for measuring health literacy in different groups, and each tool measures one aspect of health literacy Such as oral health literacy (13), adolescent health literacy (14), health literacy in women with breast cancer (15), and tools in people with chronic diseases such as diabetes (16, 17), Asthma (18), hypertension (19), health literacy competencies(20), Scale for Low Salt Consumption(21), Weight Literacy Scale (22). Abel (2014) states that, examining health literacy in different domains requires different research approaches(23). On the one hand, it is obvious that health literacy has different meanings and dimensions in different periods of life and cannot be generalized by the tools that have some medical terminology and information on some general diagnostic and therapeutic methods.
Knowing about the level of health literacy is essential to properly address the information needed by pregnant women (24). Studies of pregnant women's health literacy have been using general health literacy tools that have reported different results regarding the level of pregnant women's health literacy (25-30). Some studies have also examined the health literacy level of pregnant women using a researcher-made questionnaire and reported that only 18 to 24% of pregnant mothers had good health literacy and 31 to 34% of them had poor health literacy (8, 31). Charoghchian Khorasani et al (2017) used the maternal health literacy and pregnancy outcome questionnaire designed by Mojoyinola et al (2011) and reported that, the level of

health literacy in Iranian pregnant women is at a low level (32, 33). By examining the level of health literacy, especially at the beginning of prenatal care, we can identify those who have low levels of maternal health literacy, identify geographical areas with low maternal health literacy, and plan effective interventions in this regard.

According to searches conducted globally, there are several studies on tool design to measure maternal health literacy(33-35), but a native tool derived from a genuine research with tool design methodology (including the use of qualitative method that determines this concept from the perspective of pregnant mothers as the target group) is not available. Available studies have used general tools or researcher-made questionnaires to determine the level of maternal health literacy during pregnancy, which seem to have only assessed the health knowledge rather than health literacy. It has been emphasized that health literacy goes beyond health knowledge (36), but is closely linked to health knowledge (37, 38). Therefore, this study aimed to design and psychometrically evaluate a maternal health literacy tool during pregnancy.

Material and methods

The present study is a sequential, exploratory and mixed research that was conducted between November 2016 and August 2018. This research started with a qualitative study and continued with a quantitative study. This method was designed by Cresol and Clarke Plano as one of the five main types of mix method studies (39). This type of study is divided into two types of theory design and tool design. The present study is a tool design study (40). To build a tool, we need to understand the concept of health literacy by using the experience of participants. Having a conceptual framework is the first step in designing a tool. Therefore, the qualitative part of the study was designed and implemented to design the tool, and then its psychometric property was conformed in a quantitative study.

Step one: Qualitative study

In the qualitative phase, 23 semi-structured and in-depth interviews were conducted with 19 pregnant women referred to prenatal care clinics of the hospitals affiliated to the Tehran University of Medical Sciences from February 2016 to September 2017. Inclusion criteria were; being a pregnant woman with minimum level of education (reading and writing) and being an Iranian citizen. Interviews were conducted at the locations agreed upon by the researcher and participants (such as prenatal education classrooms in hospitals or comprehensive health centers, parks near the participants' place of residence, etc.) so that, the participants could recall their experiences more easily. In this study, 23 individual interviews were conducted with 19 pregnant women. The duration of each interview was between 30 to 130 minutes with an average of 60 minutes, depending on the amount of information, participation, and cooperation of the participants. Qualitative analysis was performed using conventional content

analysis through MAXQDA-10 software. After analyzing the findings of qualitative section and reviewing the available literature and tools, a 124-item pool was created. After reviewing the items, the members of primary research group developed a 78-item Maternal Health Literacy Assessment Tool (MHELIP) questionnaire, consisting of two sections: 1) Assessing the information related to pregnancy health, and 2) Functional health literacy. For the initial scoring, a 5-point Likert scale was used in the section of assessing the information related to pregnancy health, which ranged from I don't know at all to I know a lot (with the score of 1 to 5), and a scale of never to always (with the score of 1 to 5) was used in the section of functional health literacy. Then, the research entered the quantitative phase and psychometric of the designed tool was performed.

Step two: Tool's psychometric

1.Validity

Content validity: In order to evaluate the qualitative content validity, 10 experts in gynecology, midwifery, reproductive health, maternal and child health, health education, nursing, and health literacy were asked to review the tool and express their comments and opinions in terms of its grammar and the use of right words and phrases, and offer suggestion to add or remove items in writing. The questionnaire was then edited according to the experts' recommendations. Content validity ratio (CVR) and content validity index (CVI) were used to assess the content validity of the tool. According to the Lawche's table, to determine the content validity ratio, a group of experts consisting of 14 subjects evaluated each item with three options (essential, useful but not essential and not essential) items with the CVR of 0.51 and higher were selected for the questionnaire. In order to determine the content validity index, items were evaluated with three options (being relevance or specific, being clear, and being simple) and those with the CVI of 0.79 and higher were accepted.

Face validity. In the qualitative face validity, the difficulty of understanding the items, words and the ambiguities, and also the possibility of misinterpreting the items or the words were assessed after interviewing 15 pregnant women. For the qualitative face validity, the impact score method was used. Thus, after rating the items for importance by 15 pregnant women, items that had the score of 1.5 or above were kept.

2.Construct validity

In the present study, before determining construct validity, the initial reliability (correlation between items) was assessed. Reliability of the original questionnaire was confirmed by conducting convenience sampling among 30 pregnant women with Cronbach's alpha coefficient

of 0.94 and as a result, the tool with high reliability entered into the construct validity stage. Exploratory factor analysis approach was used for construct validity. In this section, 320 pregnant women completed the maternal health literacy questionnaire. Inclusion criteria were; having minimum level of education for reading and writing, having Iranian citizenship, having no history of medical education, and giving consent to participate in the study. The exclusion criteria included; not willing to continue with the study and having high level of stress in the last six months such as losing loved ones, etc. First, the sampling adequacy test of KMO and the Bartlett's test were used, and then analysis of main items, varimax rotation, and factor analysis of the questionnaire were determined.

3. Reliability

After confirming the validity of the questionnaire, reliability was assessed by internal consistency and test-retest methods. In the internal consistency method, consistency of the results of the tool items was investigated and then, the Cronbach's alpha coefficient was calculated for the items in each domain and the whole questionnaire. In order to perform the test-retest, 20 pregnant women completed the final questionnaire within two weeks and the infraclass correlation coefficient (ICC) was calculated.

4. Ethical statement

In order to comply with ethical considerations, permission was obtained from the Faculty of Nursing and Midwifery of Tehran University of Medical Sciences and the Deputy for Research (IR.TUMS.VCR.REC.1395.1866). Written consent was obtained for the participants and confidentiality of their personal information was maintained at all stages of the study.

Results

1. Sample characteristic

The mean age of participants in the quantitative section of this study was 28.32 ± 5.59 years and their mean gestational age was 26.75 ± 9.31 weeks. The majority of women (51.3%) were experiencing their first pregnancy and most of them (76.3%) had intended pregnancy. Most of the pregnant women had high school diploma (41.6%) and were housewife (94.1%). Most of the participants (45.6%) had the household income of between one and two million tomans and the majority of their spouse also had high school diploma (43.6%). Also, 44.4% of the participants had not attended any pregnancy training course and most of them (80.3%) had internet access.

2. Content validity

The initial questionnaire of maternal health literacy during pregnancy had 78 items. In the qualitative content validity, the items were reviewed based on the opinions of 10 experts and necessary amendments were made to the items. At this stage, 24 items were deleted. In the quantitative content validity, two indexes of content validity ratio and content validity index were calculated for 54 items. In the content validity ratio, according to the number of experts (14 persons), the minimum accepted CVR was 0.51 and in the content validity index only one item "I can solve pregnancy problems" with a score of 0.14 was removed from the original questionnaire. Finally, the questionnaire with 53 questions obtained the construct validity.

3. Face validity

In the qualitative face validity, 15 pregnant women were asked to read and provide feedback on the questionnaire, and then the proposed corrections were applied but no item was removed. In the quantitative face validity, 15 pregnant women were asked to comment on the importance of each item to determine the impact factor of each item, and all items obtained the score of above 1.5 and therefore no item was removed in this stage.

4. Construct validity

In the first step, the adequacy of the sampling was assessed for factor analysis. The statistical value of KMO = 0.905 indicated the adequacy of sampling (sample size of 320 pregnant women) for factor analysis. Bartlett's test at the significant level of 0.0001 showed no significant relationship between the items. Exploratory factor analysis was performed on 53 items in two stages. In the first stage, initial analysis of variables was performed using specific value of greater than 1 and varimax rotation, which explained 65.18% variance with 12 factors. Then, the grain size chart (Figure 1-4) was used to determine the number of factors and based on this chart, 4 factors were proposed for extraction. To ensure the structure of items, the 4 to 7 factor structure was tested with different rotations, and the best of them was identified to be the 4-factor structure. Exploratory factor analysis was performed with varimax rotation and 4 factors (Table 1). To keep the items, minimum load factor was set at 0.35. All items had the factor load of higher than 0.35. In the exploratory factor analysis phase, 5 items were removed from the maternal health literacy tool. Finally, after performing factor analysis, 4 factors with 48 items explained 46.49% of the total variance. The first factor with 21 questions explained 20.73% of the variance, second factor with 15 questions explained 12.81% of the variance, third factor with 6 questions explained 7.95% of the variance, and fourth factor with 6 questions explained 4.99% of the variance. Accordingly, they were named; " Maternal Health Knowledge", "Maternal Health Decision Making and Behavior", " Maternal Health Information Assessment" and " Maternal Health Information Search", respectively (Table 1). At the end, the questionnaire was completed with 48 items.

5. Reliability

To determine internal consistency, after ensuring construct validity, Cronbach's alpha coefficient in a sample of 320 pregnant women was 0.94 for the whole tool. However, this value varied from 0.66 to 0.94 in different dimensions. In order to determine the consistency of questionnaire in the repeatability dimension, in a group of 20 pregnant women with 2 weeks interval, the intra-cluster correlation coefficient (ICC) for the whole tool was 0.96, and this value varied from 0.74 to 0.97 in different dimensions (Table 2).

Discussion

The purpose of this study was to design and evaluate validation indexes of a questionnaire for measuring maternal health literacy in pregnant women. The initial questionnaire was designed based on data extracted from qualitative study of pregnant women, using expert opinions and reviewing existing studies in health literacy. After completing the validity and reliability stages, a maternal health literacy questionnaire was developed with 48 questions in four dimensions, which was completed by the participants. Considering that the majority of participants completed the questionnaire in approximately 15 minutes, this tool could be easily used in screening. The findings of this study showed that, the maternal health literacy questionnaire has accepted validity and reliability.

The first subscale of the maternal health literacy tool during pregnancy (MHELIP) was a 21-item construct that measured maternal health-related knowledge (questions 1 to 21). In number of maternal health literacy tools such as Chanyuan et al (2015) (35) and McCathern et al (2011), (41) there is a separate subscale called “maternal health-related knowledge”, but the content and nature of questions as well as types of responding are different in these questionnaires, and they do not have the same subject diversity and comprehensiveness as our designed tool.

The second subscale of MHELIP was the maternal health information search that consisted of 6 questions (questions 22 to 27). Although health seeking behavior is an important component of the concept of health literacy, in most health literacy questionnaires, this factor has not been considered as a separate sub-scale, even though this subscale is one of the coherent conceptual dimensions of the health literacy in pregnant women according to our qualitative study.

The third subscale of MHELIP was the maternal health information assessment that included 6 items (questions 28 to 33) that were identified in exploratory factor analysis. In the Guttersrud et al (2015) questionnaire (34), a sub-scale called; “evaluation of health information” has been added to the maternal health literacy assessment questionnaire as an independent dimension. However, the nature of the items in this questionnaire differs from our designed questionnaire.

The fourth subscale of the maternal health literacy questionnaire is the maternal health decision making and behavior that included 15 items (questions 34 to 48). Pregnancy is an important period, in which the mother needs to take appropriate health-related behaviors and decision-making in order to maintain her health and the health of her fetus. There are no such sub-scales in other maternal health literacy questionnaires and questions that are close to ones in this section fall into the general scope of functional health literacy and are not comprehensive as the questions in our designed tool. On the one hand, in this section of our designed tool, there are some questions about interacting with health care professionals, and having appropriate attention and behavior in regard to sharing of accurate information with other pregnant mothers, which do not exist in other maternal health literacy tools.

Studies have suggested that health literacy is a social structure that should be considered as a multidimensional hidden construct(42). Therefore, to measure it, we need different tools for different areas. Also, since a single definition of health literacy cannot be provided, it cannot be measured by a single tool, as the complexity of health literacy requires a multidimensional tool. The literature review showed that Chanyuan et al (2015) (35), McCathern (2011) (41), Kharazi et al (2016) (43), and Guttersrud et al (2015) (34) have designed or validated some tools to assess health literacy during pregnancy, but in the present study, items of the designed tool were extracted from a qualitative study in which, the concept of maternal health literacy was explained according to the understanding and experience of pregnant women. None of the tools available in this regard has highlighted the importance of using the experiences of pregnant women as their primary target group.

One of the most important features of a holistic tool is its comprehensiveness that covers all dimensions of a concept. In the present study, four domains extracted in the qualitative phase were also expressed during factor analysis, while in other tools the number of factors is less and even their items do not have the proper consistency with the titles of above-mentioned dimensions. On the other hand, although the concept of health literacy begins with the information seeking behavior, the subscale of maternal health information search has not been considered as a separate and independent dimension. However, in our designed tool, an independent dimension had acceptable and stable number of items and appropriate reliability.

In the designing of the tool, we tried to make the items of each domain truly genuine, have suitable comprehensiveness and be specific for pregnancy period, which were confirmed in the factor analysis. Meanwhile in tools such as the one designed by Kharrazi et al (2016) (43), the dimension of speech and listening perception includes items that are quite general, such as I can read and write, etc. In the designed questionnaire, based on the fact that during pregnancy the mothers were seeking information about postpartum health, the information on postpartum health was defined in terms of mother and child health. But since the tool was

intended to be a pregnancy specific tool and the questions would be able to cover the pregnancy itself, only two general items were measuring the maternal knowledge about postpartum health of mother and infant. In the Chanyuan (2015) tool (35), the dimension of knowledge and the concept of basic dimensions of maternal health included items that covered not only pregnancy but also postpartum, and in the domain of lifestyle and behavioral dimensions (which regardless of the title of domain and content of items, examine maternal knowledge), only one item was related to the pregnancy and the rest were related to the postpartum period. Also, in their tool the dimension of basic skills during pregnancy examines the ability to read a book, attend a pregnancy class, have a scheduled appointment, and have at least five visits to prenatal clinic, which only the item of "I regularly attend the scheduled visits" has been designed as one of the items of the questionnaire. This is while the decision-making and behavior construct of the designed questionnaire examines the health literacy of the individual in this dimension in a broader way and this leads to the better understanding of individual's situation.

In the Guttersrud et al (2015) questionnaire(34), the domain of one's perception of competence and adaptation skills includes questions that examine information search, information comprehension, ability to transfer information to others, ability to recognize the risk symptoms, follow-up and readiness for childbirth, which are similar to the assessment, decision making and behavior, and the knowledge sections of the designed questionnaire. However, despite this similarity, the nature of questions in the two tools is not the same. In the Guttersrud et al (2015) tool (34), the information assessment questions include separation of right from wrong, easy recall of previous information, independence in following recommendations, being active, social participation like pre-pregnancy period, and ability to care for own self and the infant. The questions in the above tool are a mixture of questions on various aspects of health literacy and it is true that the title of this construct is similar to our designed tool, but the nature of its questions are different from the tool designed in the present study. Perhaps the concept of health literacy is also different in the two tools. This is the strength of our designed tool to report health literacy as a whole, and also as dimensions that are completely independent of one another, which can confirm the importance of a mixed exploratory study that could complete the entire psychometric evaluation process, including construct validity.

The tool designed to measure pregnancy knowledge covers issues needed by pregnant women. Questions in this tool are suitable for the general public of pregnant mothers while in other tools such as Chanyuan (2015) tool (35), Knowledge questions may not have a high difficulty coefficient and low discriminate coefficient for identifying individuals with different levels of health literacy. But, this difference in the design of questions may be due to the knowledge of researcher about the community of pregnant woman and his/her expectation about the knowledge debate in pregnant women of that community. Also, the prenatal care

questionnaire used by McCathern (41)(2011) had 5 items with 4-option answer in the field of prenatal care knowledge, and although this type of questions can measure the level of knowledge properly, it may be possible to ask if these tools were qualitatively faced validated by pregnant mothers, would they still obtain an acceptable score of importance? Failure to perform quantitative face validity by those who are the most important target group of the questionnaire is one of the weaknesses of this questionnaire and similar questionnaires that have been considered in the designed tool. Designing a maternal health literacy questionnaire for pregnant women in Iran is the first fundamental step in examining the health literacy of Iranian pregnant women and it is hoped that, this tool can be an incentive to initiate extensive and substantive research into the importance and dimensions of health literacy in pregnant women.

Conclusion

The maternal health literacy assessment tool during pregnancy is a valid and reliable tool and can be used in future studies to measure pregnant women's health literacy.

Acknowledgments

This study is part of a PhD dissertation at Tehran University of Medical Sciences with code of ethics IR.TUMS.VCR.REC.1395.1866. We would like to thank and appreciate all the experts and participants who helped us with this study patiently.

Competing interests

The authors declare that they have no competing interests.

Authors' contributions

ST supervised all stages of the study, analyzed and interpreted the data and wrote the manuscript. ZT and MT participated in the planning and supervised all stages of the study. ZM and AT participated in analyzed and interpreted the data. NSH and ZS participated in wrote the manuscript.

All authors critically reviewed and revised the manuscript for important contents. All the authors have read and approved the final manuscript.

Funding

This study is part of the PhD thesis in Tehran University of Medical Sciences, Iran. But this institution did not take part in study planning, data collection, data analysis and manuscript writing.

Availability of data and materials

The datasets used and/or analyzed during the current study are available from the corresponding author on reasonable request.

Consent to publish

The manuscript does not include any individual person's data; hence consent to publish is not applicable.

Reference

1. American College of Obstetrics & Gynecology. 2003; Guidelines of ACOG.

2. Ajorloo A, Shakibaei P, Investigation of Effects of Aerobic Exercise on Pregnancy and Its Circumstances. "Hayat" Nursing & Midwifery,Tehranuniversity Of Medical Sciences.2006;12(3): 35-41.

3. Emamiafshar N, Jalilvand P, Delavar B, Radpouyan L, Azemikhah A, Valafar S. National Maternal Surveillance System. 1st Ed. Tehran: Tandis. 2006.

4. Tabandeh A KE. Effects of Maternal Body Mass Index and Weight Gain During Pregnancy On the Outcome of Delivery, Journal Gorgan University of Medical Science. 2007;9(1): 20-24.

5. Moos Mk.Prenatal Care: Limitations And Opportun Ities. Jognn .2006: 35(2): 278-85.

6. Moynihan A. The Association Of Maternal Health Literacy Levels And Preterm Birth, Dissertation Submitted In Partial Fulfillment Of The Requirements For The Degree Of Doctor Of Philosophy. 2015.

7. Bennett I. Breaking It Down: Patient-Clinician Communication and Prenatal Care Among African American Women Of Low And Higher Literacy. Annals Of Family Medicine .2006; 4: 334-340.

8. Kohan S, Ghasemi S, Dodangesh M. Association Between Maternal Health Literacy And Prenatal Care And Pregnancy Outcome. Iranian Journal Of Nursing And Midwifery Research .2007;12 (4): 146-152.

9. Renkert S, Nutbeam D. Opportunities To Improve Maternal Health Literacy Through Antenatal Education: An Exploratory Study. Oxford Journals Medicine & Health, Health Promotion International .2001;16(4): 381-388.

10. Lori Chyd J R, Ackah J V, Adanu R MK. Examining Antenatal Health Literacy In Ghana . J Nurs Scholarsh November. 2014; 46(6): 432-440.

11. Pembe Ab, Carlstedt A, Urassa Dp, Lindmark G, Nystrom L, Darj E. Quality Of Antenatal Care In Rural Tanzania: Counselling On Pregnancy Danger Signs. Bmc Pregnancy & Childbirth. 2010; 10(35): 1–7.

12. Haun JN,Valerio M A, Mccormack L A, Sørensen K, Paasche-Orlow M K. Health Literacy Measurement: An Inventory And Descriptive Summary Of 51 Instruments, Journal Of Health Communication: International Perspectives.2014;19: 302-333.

13. Naghibi Sistan M Ma, Yazdani R , Murtomaa H. New Oral Health Literacy Instrument For Public Health: Development And Pilot Testing. Journal Of Investigative And Clinical Dentistry. 2014;5: 313-21.

.14. Ghanbari S, Ramezankhani A, Montazeri A,Mehrabi Y . Health Literacy Measure for Adolescents (HELMA): Development andPsychometric Properties. PLoS ONE. 2016;11(2): 149-202. doi:10.1371/journal.pone.0149202

15. Khalili S, Tavousi M, Moghaddam Banaem L. Health Literacy for women with breast cancer (HELBA): development and psychometric properties. Payesh. 2017; 16 (3) :359-366 URL: http://payeshjournal.ir/article-1-109-fa.html. [in Persian]

16. Nath CR, et al. Development and validation of a literacy assessment tool for persons with diabetes. The Diabetes Educator. 2001. 27(6): 857-864.

17. Huizinga MM, et al. Development and validation of the Diabetes Numeracy Test (DNT). BMC health services research. 2008. 8(1): 96.

18. Apter AJ, et al. Asthma numeracy skill and health literacy. Journal of Asthma. 2006. 43(9): 705-710.

19. Kim MT, et al. Development and validation of the high blood pressure-focused health literacy scale. Patient education and counseling. 2012. 87(2): 165-170.

20. Chau P, Leung AYM, Li HLH, Sea M, Chan R, Woo J. Development and Validation of Chinese Health Literacy Scale for Low Salt Consumption - Hong Kong Population (CHLSalt-HK). PLoS ONE. 2015;10(7): e0132303. doi:10.1371/journal. pone.0132303.

21.Chang L-C, Chen Y-C, Liao L-L, Wu FL, Hsieh P-L, Chen H-J. Validation of the instrument of health literacy competencies for Chinese-speaking health professionals. PLoS ONE .2017;12(3): e0172859. doi:10.1371/journal. pone.0172859

22.Wang ML, Little TV, Frisard C, Borg A, Lemon SC, Rosal MC. Development and validation of a Weight Literacy Scale in English and Spanish. PLoS ONE. 2018;13(10): e0204678. https://doi.org/10.1371/journal.pone.0204678

23. Abel T, Hofmann K, Ackermann S, Bucher S, Sakarya S. Health Literacy Among Young Adults: A Short Survey Tool For Public Health And Health Promotion Research. Health Promot Int. 2015 Sep; 30(3): 725-35.

24. Walfisch A, Sermer C, Matok I, Einarson A, Koren G. Perception Of Teratogenic Risk And The Rated Likelihood Of Pregnancy Termination: Association With Maternal Depression. Canadian Journal Of Psychiatry. 2011; 56(12): 761-767.

25. Sheih C, Mays R, Mcdaniel A, Yu J.Health Literacy And Its Association With The Use Of Information Sources And With Barriers To Information Seeking In Clinic-Based Pregnant Women. Health Care For Women International. 2009; 30: 971-988.

26.Baghaei R, Najarzadeh M, Saei M, Mohamadi N. FUNCTIONAL HEALTH LITERACY IN PREGNANT WOMEN IN HEALTH CENTERS OF URMIA CITY- 2015. J Urmia Nurs Midwifery Fac. 2017; 15 (5) :368-375.URL: http://unmf.umsu.ac.ir/article-1-3179-fa.html. [in Persian]

27. Cho RN, Plunkett BA, Wolf MS, Simon CE, Grobman WA. Health literacy and patient understanding of screening tests for aneuploidy and neural tube defects. Prenat Diagn. 2007;27(5): 463– 467.

28.Safari Morad Abadi A, Agha Molaei T, Ramezankhani A, Dadipoor S. The Health Literacy of Pregnant Women in Bandar Abbas, Iran. Sjsph. 2017; 15 (2) :121-132. URL: http://sjsph.tums.ac.ir/article-1-5524-fa.html. [in Persian]

29. Ghanbari S, Majlessi F, Ghaffari M, Mahmoodi Majdabadi M. Evaluation of health literacy of pregnant women in urban health centers of Shahid Beheshti Medical University . Daneshvar Medicine. 2012; 19 (97) :1-12. URL: http://daneshvarmed.shahed.ac.ir/article-1-480-fa.html. [in Persian]

30. Izadirad H, Niknami Sh, Zareban I, Tavousi M. Health literacy and prenatal care adequacy index on the outcome of birth weight in
pregnant women in Balochistan, Iran. Journal of the Iranian Institute for Health Sciences Research. 2018; 17(2):191-198. [in Persian]

31. Amiresmaili M, Nekoei Moghadam M ,Saberi anari SH, Sadeghi A, Saber M,Taheri G,Hosseini SH, Rezazadeh J. Study of health literacy level of women referring tohealth centers-2010. Journal of North Khorasan University of Medical Sciences. 13;5(Student Research Committe Supplementary):1079. [in Persian]

32. Charoghchian Khorasani E PN, Esmaily H. Relations between Breast feeding Self-efficacy and Maternal Health Literacy among Pregnant Women. Evidence Based Care Journal. 2017; 6 (4): 18-25. [in Persian]

33. Mojoyinola JK. Influence of Maternal Health Literacy on Healthy Pregnancy and Pregnancy Outcomes of Women Attending Public Hospitals in Ibadan OS, Nigeria. Afr Res Rev. 2011; 5(3): 28-39.
34. Guttersrud Ø, Naigaga MDAS , PettersenKS .Measuring maternal health literacy in adolescents attending antenatal care in Uganda. Exploringthe dimensionality of the "health liter-acy" concept studying a compositescale. Journal of Nursing Measure-ment. 2015;23(2):.50E-66.

35. Chanyuan D Jy, Linyong X, Xiao Qi. Item Selection For Perinatal Maternal Health Literacy Scale. J Cent South Univ (Med Sci). 2015; 40(5): 558-563.

36. Ratzan Sc. Health Literacy: Communication for The Public Good. Health Promotion International. 2001; 16(2): 207–214.

37. Dennison Cr, Mcentee Ml, Samuel L, Et Al. Adequate Health Literacy Is Associated With Higher Heart Failure Knowledge And Self-Care Confidence In Hospitalized Patients. J Cardiovasc Nurs. 2011; 26(5): 359–367.

38. Macabasco-O'connell A, Dewalt Da, Broucksou Ka, Et Al. Relationship Between Literacy,Knowledge, Self-Care Behaviors, And Heart Failure-Related Quality Of Life Among Patients With Heart Failure (Published Online Ahead Of Print March 3, 2011). J Gen Intern Med. 2011; 26(9): 979– 986.

39. Creswell JW, Plano Clark VL. Designing and Conducting Mixed Methods Research. 2007.

40. Creswell J W, Clark V L. P. Designing and Conducting Mixed Methods Research, SAGE.2014.

41. McCathern, Rhonda M.Exploring the Predictive Relationship Between General Health Literacy Levels and Prenatal Care Health Literacy Levels. Seton Hall University Dissertations and Theses (ETDs). 2011.

42. Pleasant A, Mck inney J, Rikard R. Health Literacy measurement: A propsed research agenda. Journal of health communication. 2011; 16(sup3): 11-21.

43. Kharazi S ،Peyman N ، Esmaily H. Association between maternal health literacy level with pregnancy care and its outcomes. The Iranian Journal of Obstetrics, Gynecology and Infertility.19(37).40-50. 10.22038/ijogi.2016.8187. [in Persian]

Table 1: The 4-factor construct and factor load of each item

Item	First factor	Second factor	Third factor	Fourth factor
Knowing about natural physical changes during pregnancy	**0.510**	0.157	0.287	0.234
Understanding the natural psychological changes during pregnancy	**0.521**	0.149	0.282	0.148
Knowing about proper nutrition during pregnancy	**0.605**	0.216	0.191	0.059
Knowing how to respect personal hygiene	**0.599**	0.174	0.319	0.052
Knowing the basics of pregnancy activity and situation	**0.703**	0.171	0.107	0.042
Knowing about proper exercise during pregnancy	**0.590**	0.079	0.187	0.001
Knowing about pregnancy supplements (vitamins)	**0.665**	0.171	0.107	0.042
Knowing the appropriate referral timing for pregnancy examinations (visits)	**0.644**	0.168	0.207	0.025
Understanding diagnostic examination (ultrasound and tests) of maternal and fetal health in pregnancy	**0.707**	0.175	0.143	0.053
Knowing about the acceptable and normal amount of weight gain during pregnancy	**0.717**	0.155	0.131	0.007
Knowing about common pregnancy problems such as nausea, vomiting, lower back pain	**0.681**	0.125	0.105	0.069
Know about injecting safe (authorized) vaccines during	**0.681**	0.133	0.022	0.097

pregnancy				
Knowing about the proper sexual relation during pregnancy	**0.719**	0.239	0.060	0.087
Knowing the normal number of fetal movements	**0.696**	0.158	0.012	0.117
Know the factors affecting fetal health such as photography, medications, chemicals such as botox, etc	**0.648**	0.101	0.134	0.105
Knowing about risk signs in pregnancy	**0.738**	0.102	0.020	0.109
Knowing about pregnancy disease symptoms such as gestational diabetes, high blood pressure in pregnancy and other diseases	**0.714**	0.074	0.110	0.215
Knowing about childbirth such as the advantages and disadvantages of each of the natural delivery methods and cesarean section and their associated care	**0.630**	0.097	0.064	0.238
Knowing about the methods of pain relief in virginal delivery	**0.544**	0.019	0.124	0.165
Knowing about neonatal and infant care in the postpartum period	**0.596**	0.183	0.143	0.316
Knowing about required postpartum care of mother	**0.653**	0.130	0.080	0.289
Acquiring information from written materials such as books, educational notes, pamphlets and medication brochures	0.102	0.205	0.263	**0.412**
Acquiring information from radio and television	0.157	0.046	0.001	**0.467**
Acquiring information from internet sources such as websites, instagram and telegram	0.152	0.058	0.061	**0.767**
Acquiring information from other pregnant women	0.016	0.041	0.199	**0.580**
Acquiring information from family, friends and acquaintances	0.068	0.000	0.033	**0. 590**
Acquiring information from healthcare professionals such	0.224	0.302	0.115	**0.378**

as a physician or midwife				
Easy to read and pronounce pregnancy-related vocabulary from information sources such as books, educational booklets, internet, telegram and instagram	0.206	0.122	**0.734**	0.025
Understand information obtained from different sources of information	0.287	0.296	**0.611**	0.032
Getting familiar with the reliable and verified sources to get the right information	0.233	0.044	**0.733**	0.113
Asking the doctor or midwife to make sure information is reliable	0.144	0.205	**0.385**	0.293
Evaluating the accuracy of pregnancy-related information obtained from online sources such as websites, instagram and telegram	0.098	0.075	**0.758**	0.239
Evaluating the accuracy of pregnancy-related information obtained from friends and relatives	0.150	0.214	**0.414**	0.084
Ability to manage and control physical and psychological changes in pregnancy	0.329	**0.539**	0.157	0.205
Implement a proper diet for pregnancy	0.244	**0.591**	0.030	0.072
Implement necessary measures for personal hygiene during pregnancy	0.224	**0.575**	0.105	0.033
Adhere to the principles of activity and proper condition during pregnancy	0.352	**0.496**	0.005	0.040
Taking pregnancy supplements as directed by doctor or midwife	0.101	**0.546**	0.044	0.079
Consult with the doctor or midwife for taking any type of medication during pregnancy (chemical and herbal)	0.036	**0.615**	0.214	0.086
Attending for prenatal care (examinations) as scheduled	0.138	**0.652**	0.041	0.016
Performing ultrasound and tests in pregnancy recommended by healthcare professionals such as doctor	0.046	**0.737**	0.111	0.017

or midwife				
Monitoring the weight gain during pregnancy	0.079	**0.590**	0.053	0.012
Using appropriate methods of sexual relation during pregnancy	0.161	**0.557**	0.014	0.10
Avoiding harmful practices in pregnancy	0.177	**0.528**	0.024	0.038
Seeing the doctor or midwife as soon as possible when any signs of danger in pregnancy is observed	0.072	**0.645**	0.001	0.049
Asking the doctor or midwife for further explanation if the information and recommendations are not clear enough	0.051	**0.629**	0.216	0.253
Participate in decision making about pregnancy issues with the doctor or midwife (providing personal opinions)	0.100	**0. 476**	0.277	0.207
Paying attention to the accuracy and appropriateness of information given to other pregnant women	0.043	**0.442**	0.276	0.305
Rotation sums of squared loadings	**20.73**	**33.55**	**41.50**	**46.49**
Percentage of data dispersion by each dimension	**20.73**	**12.81**	**7.95**	**4.99**
Percentage of variance coverage of total changes	**49.46**			
**factor1:** Maternal Health Knowledge ,**factor2:** Maternal Health Decision Making and Behavior ,**factor3:** Assessment of Maternal Health Information , **factor4:** Search for maternal health information				

Table 2: The scores of Cronbach's alpha coefficient, ICC constructs and the whole MHELIP tool

No	Construct	Cronbach's alpha coefficient	*ICC
1	Maternal Health Knowledge	0.94	0.97
2	Search for maternal health information	0.66	0.74
3	Assessment of Maternal Health Information	0.79	0.86
4	Maternal Health Decision Making and Behavior	0.87	0.92
5	Overall reliability of the tool	0.94	0.96
***ICC: Infraclass correlation coefficient**			

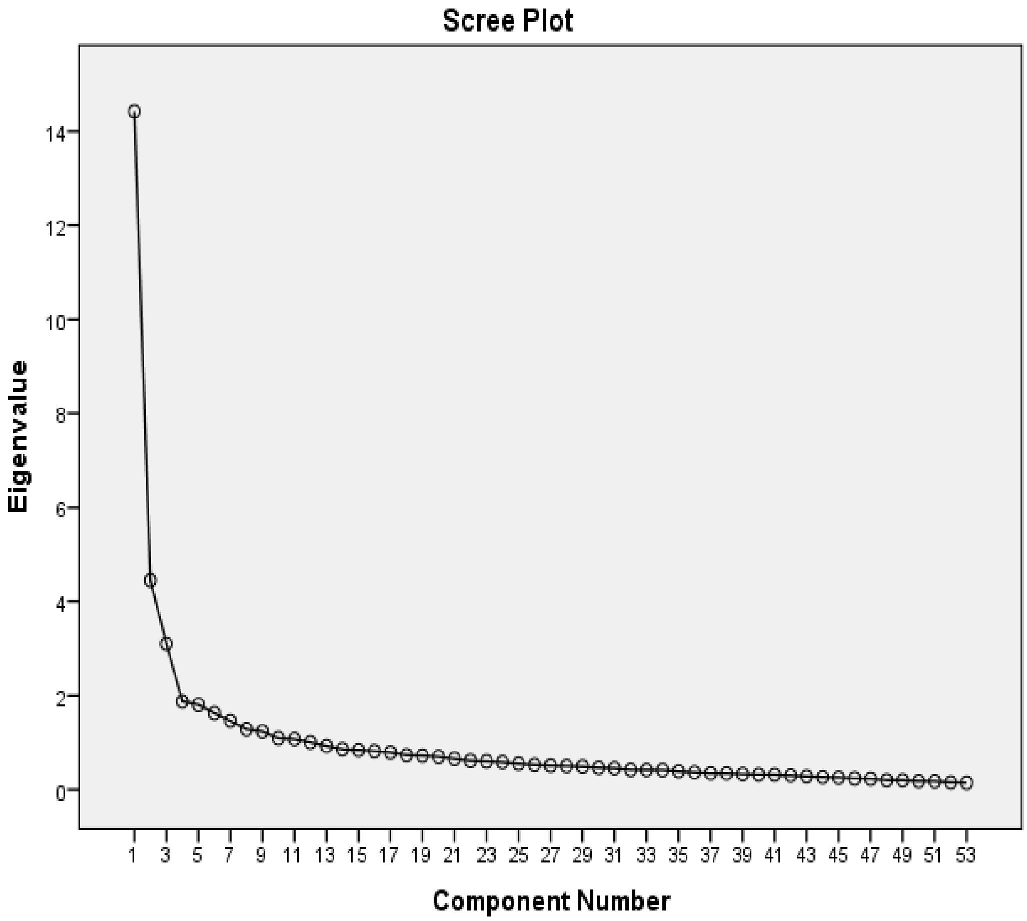

Figure 1: Scree Plot
