# Supplementary material for: Development and psychometric properties of maternal health literacy inventory in pregnancy
Source: PLoS One. 2020 Jun 11;15(6):e0234305. doi: 10.1371/journal.pone.0234305 (PMC7289409; doi:10.1371/journal.pone.0234305)
Supplement: Attachment — Submitted filename: PONE-D-19-24536_reviewer comments.docx [file pone.0234305.s007.docx]

The aim of this study was to develop a tool to measure maternal health literacy during pregnancy and test its psychometric properties. This is an interesting and timely study particularly in the low and middle-income country context. I have few comments for the authors to consider:

1. I would suggest authors to rephrase their study objective mentioned in the abstract (perhaps in the introduction too).
2. In the last paragraph of the introduction, authors have mentioned: “*but a native tool derived from a genuine research”,* I would encourage authors to replace the word ‘genuine’ from ‘original’ or another appropriate word.
3. When was this study actually started- November 2016 (mentioned in material and methods) / February 2016 (mentioned in qualitative study)? Please correct the inconsistency.
4. How did authors actually recruit participants (19 pregnant women) for the qualitative study (over 1.5+ years)? More details are needed.
5. I suggest authors to state the number of semi-structured and in-depth interviews conducted separately.
6. How did authors conduct 23 individual interviews with 19 pregnant women?
7. Please provide your interview guide as an appendix.
8. In my opinion, below chunk of text needs to be in the result section.

*After analyzing the findings of qualitative section and reviewing the available literature and tools, a 124-item pool was created. After reviewing the items, the members of primary research group developed a 78-item Maternal Health Literacy Assessment Tool (MHELIP) questionnaire, consisting of two sections: 1) Assessing the information related to pregnancy health, and 2) Functional health literacy. For the initial scoring, a 5-point Likert scale was used in the section of assessing the information related to pregnancy health, which ranged from I don't know at all to I know a lot (with the score of 1 to 5), and a scale of never to always (with the score of 1 to 5) was used in the section of functional health literacy.*

9. How did authors compute Content Validity Ratio (CVR) and Content Validity Index (CVI)?
10. I suggest authors to expand on the ‘impact score method’ in the face validity section.
11. Please provide references for Content Validity Ratio (CVR) and Content Validity Index (CVI), Lawche’s table and impact score method.
12. How did authors collect data for psychometric analysis, e.g. sample selection, who collected the data, setting, etc., and please explain?
13. Perhaps, authors could also use a flow chart to summarize/simplify the entire psychometric evaluation process and scale item selection.
14. How did authors identify participants having minimum level of education for reading and writing?
15. Eligible participants who are not willing to participate to a study are generally called as non-respondents. However, authors have listed them under the exclusion criteria here. In this case, there is a possibility of having a systematic difference between respondents and non-respondents.
16. I suggest to present participant characteristics of the qualitative study and then consider addressing reviewer comment 8.
17. Please revisit your interpretation on Bartlett’s test of sphericity. <https://www.ibm.com/support/knowledgecenter/SSLVMB_26.0.0/statistics_casestudies_project_ddita/spss/tutorials/fac_telco_kmo_01.html>
18. There is an inconsistency of figure labelling [e.g. Figure 1-4 (in text)]
19. Suggest to report Cronbach’s alpha and ICC values with 95% confidence intervals.
20. Please highlight the main strengths and limitations of this study.
21. Please correct the typos and inconsistencies in the reference section, e.g. no 5.
