# Supplementary material for: Development and psychometric properties of maternal health literacy inventory in pregnancy
Source: PLoS One. 2020 Jun 11;15(6):e0234305. doi: 10.1371/journal.pone.0234305 (PMC7289409; doi:10.1371/journal.pone.0234305)
Supplement: Attachment — Submitted filename: reply comment.docx [file pone.0234305.s008.docx]

The aim of this study was to develop a tool to measure maternal health literacy during pregnancy and test its psychometric properties. This is an interesting and timely study particularly in the low and middle-income country context. I have few comments for the authors to consider:

**dear reviewer, thank you for your valuable comments.**

1. I would suggest authors to rephrase their study objective mentioned in the abstract (perhaps in the introduction too). Applied to the article. 33-35 line, 114-116 line.
2. In the last paragraph of the introduction, authors have mentioned: “*but a native tool derived from a genuine research”,* I would encourage authors to replace the word ‘genuine’ from ‘original’ or another appropriate word. Applied to the article. 108 line
3. When was this study actually started- November 2016 (mentioned in material and methods) / February 2016 (mentioned in qualitative study)? Please correct the inconsistency. Applied to the article.128 line and 150-153 line.
4. How did authors actually recruit participants (19 pregnant women) for the qualitative study (over 1.5+ years)? More details are needed. Applied to the article.128line

The first phase (qualitative study) of the study was performed from February 2016 to September 2017, about Seven- Eight months.

5. I suggest authors to state the number of semi-structured and in-depth interviews conducted separately. Applied to the article.128-131 line., 135-137 line
6. How did authors conduct 23 individual interviews with 19 pregnant women? Applied to the article135-137 line
7. Please provide your interview guide as an appendix. Applied in support information
8. In my opinion, below chunk of text needs to be in the result section. Applied to the article. 210-218 line

*After analyzing the findings of qualitative section and reviewing the available literature and tools, a 124-item pool was created. After reviewing the items, the members of primary research group developed a 78-item Maternal Health Literacy Assessment Tool (MHELIP) questionnaire, consisting of two sections: 1) Assessing the information related to pregnancy health, and 2) Functional health literacy. For the initial scoring, a 5-point Likert scale was used in the section of assessing the information related to pregnancy health, which ranged from I don't know at all to I know a lot (with the score of 1 to 5), and a scale of never to always (with the score of 1 to 5) was used in the section of functional health literacy.*

9. How did authors compute Content Validity Ratio (CVR) and Content Validity Index (CVI)? Applied to the article. 180-190 line
10. I suggest authors to expand on the ‘impact score method’ in the face validity section. Applied to the article. 170-175 line
11. Please provide references for Content Validity Ratio (CVR) and Content Validity Index (CVI), Lawche’s table and impact score method. Applied to the article. 163-166 line
12. How did authors collect data for psychometric analysis, e.g. sample selection, who collected the data, setting, etc., and please explain? Applied to the article. 180-190 line
13. Perhaps, authors could also use a flow chart to summarize/simplify the entire psychometric evaluation process and scale item selection. Applied to the article.178 and 558 line.

How did authors identify participants having minimum level of education for reading and writing? Applied to the article. 178line. When, the samples invited to the study, the researcher asked them about Ability to read and write.

14. Eligible participants who are not willing to participate to a study are generally called as non-respondents. However, authors have listed them under the exclusion criteria here. In this case, there is a possibility of having a systematic difference between respondents and non-respondents. Applied to the article. First 189line. The sentence changed :Incomplete questionnaire completion
15. I suggest to present participant characteristics of the qualitative study and then consider addressing reviewer comment 8. Applied to the article. 207-218 line
16. Please revisit your interpretation on Bartlett’s test of sphericity. <https://www.ibm.com/support/knowledgecenter/SSLVMB_26.0.0/statistics_casestudies_project_ddita/spss/tutorials/fac_telco_kmo_01.html>. Applied to the article.244-245 line
17. There is an inconsistency of figure labelling [e.g. Figure 1-4 (in text)] Applied to the article. 248 line.
18. Suggest to report Cronbach’s alpha and ICC values with 95% confidence intervals. Applied to the article. 264-268 line
19. Please highlight the main strengths and limitations of this study. Applied to the article. 374-385line
20. Please correct the typos and inconsistencies in the reference section, e.g. no 5. Applied to the article. 413-548 line
