# Supplementary material for: Development and psychometric properties of maternal health literacy inventory in pregnancy
Source: PLoS One. 2020 Jun 11;15(6):e0234305. doi: 10.1371/journal.pone.0234305 (PMC7289409; doi:10.1371/journal.pone.0234305)
Supplement: Attachment — Submitted filename: Response to Reviewers.docx [file pone.0234305.s009.docx]

**dear reviewer, thank you for your valuable comments**

Reviewer #1: The manuscript has been revised accordingly. It has good merit for publication. I recommend for acceptance. Thanks a lot.

Reviewer #2: Line 124, I suggest the authors to mention their study period as February 2016 to August 2018. Applied to the article. 120 line

Line 140-142-did the authors exclude four interviews (first round) during the analysis since they have repeated interviews with four women? Please clarify. Applied to the article. 131-132 line. In total, we interviewed 19 pregnant mothers individually, deeply and semi-structured. In 4 of these individuals (19 pregnant) the interview lasted and at another time the interview continued to complete the accuracy. The sentence was corrected to clear up the ambiguity

5. I suggest authors to state the number of semi-structured and in-depth interviews
conducted separately. Applied to the article.128-131 line., 135-137 line.
No information is provided on the number of semi-structured interviews and in-depth interviews conducted. In the abstract authors have only mentioned about in-depth interviews. Applied to the article. 38-39 line 131-132 line. In total, we interviewed 19 pregnant mothers individually, deeply and semi-structured. In 4 of these individuals (19 pregnant) the interview lasted and at another time the interview continued to complete the accuracy. The sentence was corrected to clear up the ambiguity


18. Suggest to report Cronbach’s alpha and ICC values with 95% confidence intervals.
Applied to the article. 264-268 line
This comment has been addressed by the authors incorrectly.
In the manuscript text and Table 3, 95% confidence intervals are not presented for Cronbach’s alpha and ICC values. Please mention the values. Applied to the article. 597 line

20. Please correct the typos and inconsistencies in the reference section, e.g. no 5. Applied
to the article. 413-548 line . Applied to the article. 424-559 line

p value of Bartlett’s test should be written as follows: (p value <0.001). Applied to the article256 line


Suggest to revise the manuscript with a native English speaker. Edited by Native
